# Supplementary material for: Proteome-Wide Identification and Comparison of Drug Pockets for Discovering New Drug Indications and Side Effects
Source: Molecules. 2025 Jan 10;30(2):260. doi: 10.3390/molecules30020260 (PMC11767986; doi:10.3390/molecules30020260)
Supplement: Supplementary file 1 [file molecules-30-00260-s001.zip › Supplementary Figure.pdf]

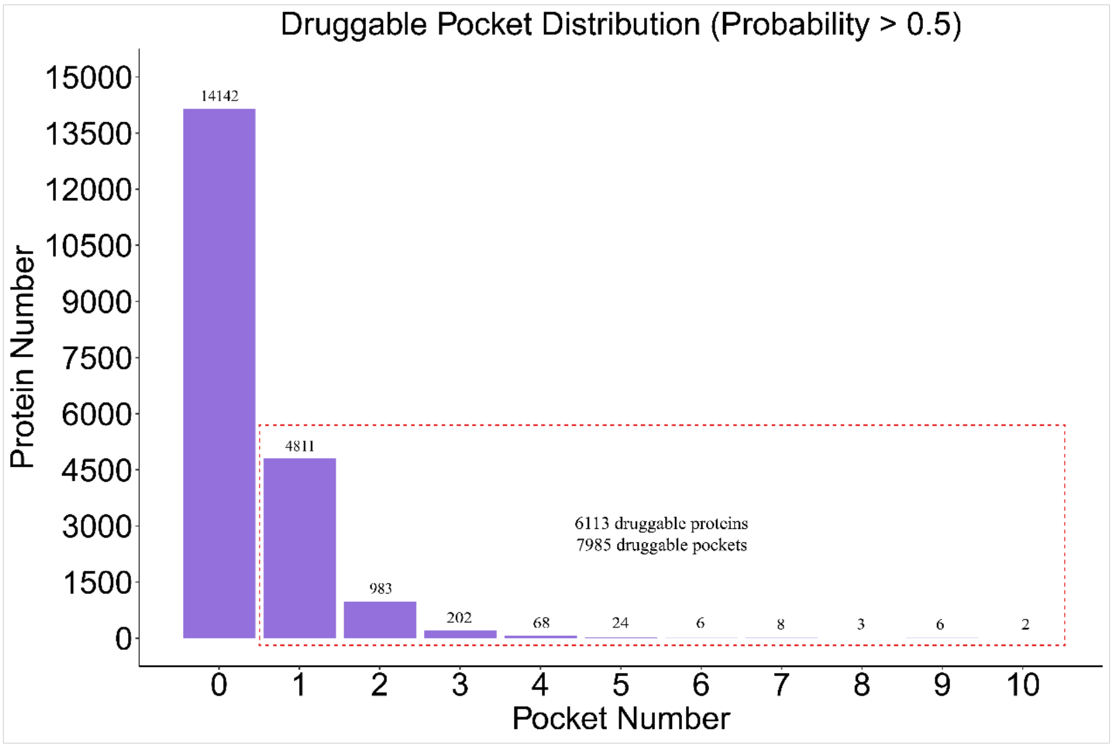

Figure S1. Bar chart of druggable pockets and druggable proteins quantities in the human proteome predicted by P2Rank, 7,985 druggable pockets and 6,113 druggable proteins were identified.

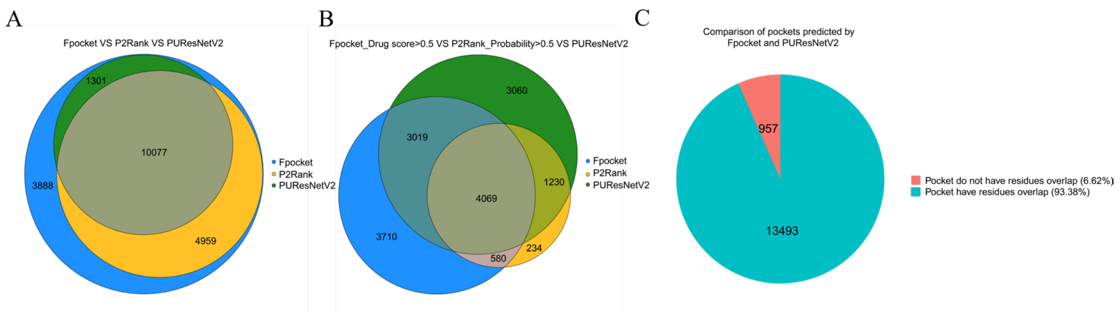

Figure S2. The number of druggable proteins predicted by Fpocket, P2Rank and PUResNetV2. (A) The number of druggable proteins predicted by Fpocket, P2Rank and PUResNetV2 without screen, all the druggable proteins predicted by PUResNetV2 can be predicted by Fpocket. (B) The number of druggable proteins predicted by Fpocket, P2Rank, and PUResNetV2 after screening, Fpocket and PUResNetV2 predicted more than 7,000 druggable protein intersections. (C) The number of pockets predicted by PUResNetV2 intersecting with those predicted by Fpocket.

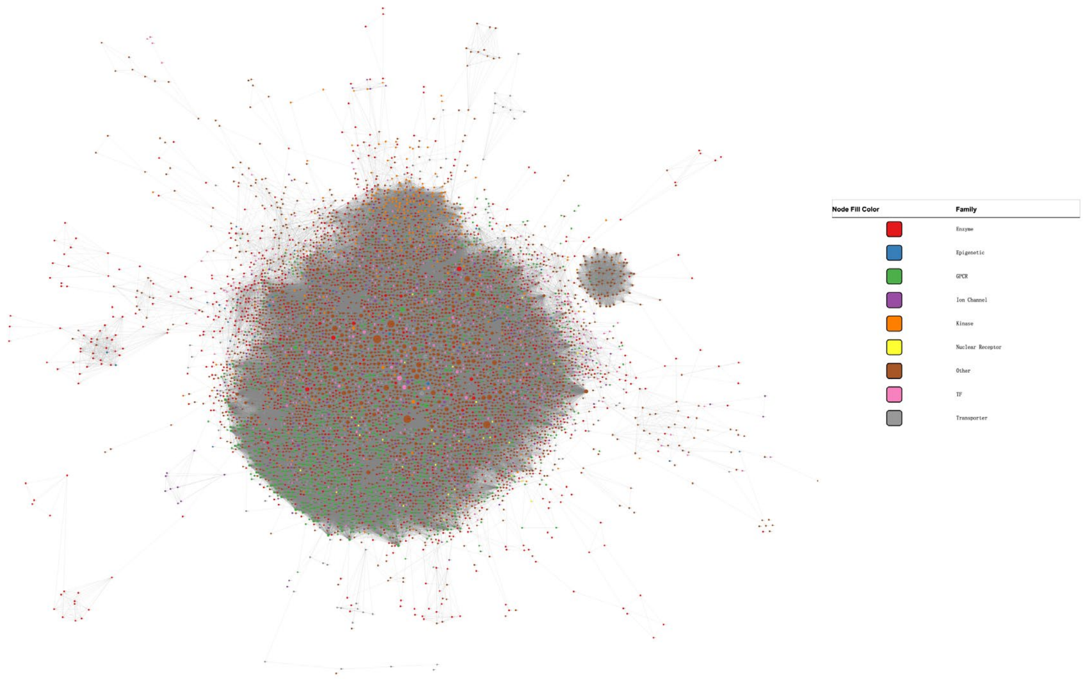

Figure S3. Network of entire human proteome similar pocket pairs. Node represent druggable pocket, the larger the node size, the more similar pocket pairs are formed with other pockets.

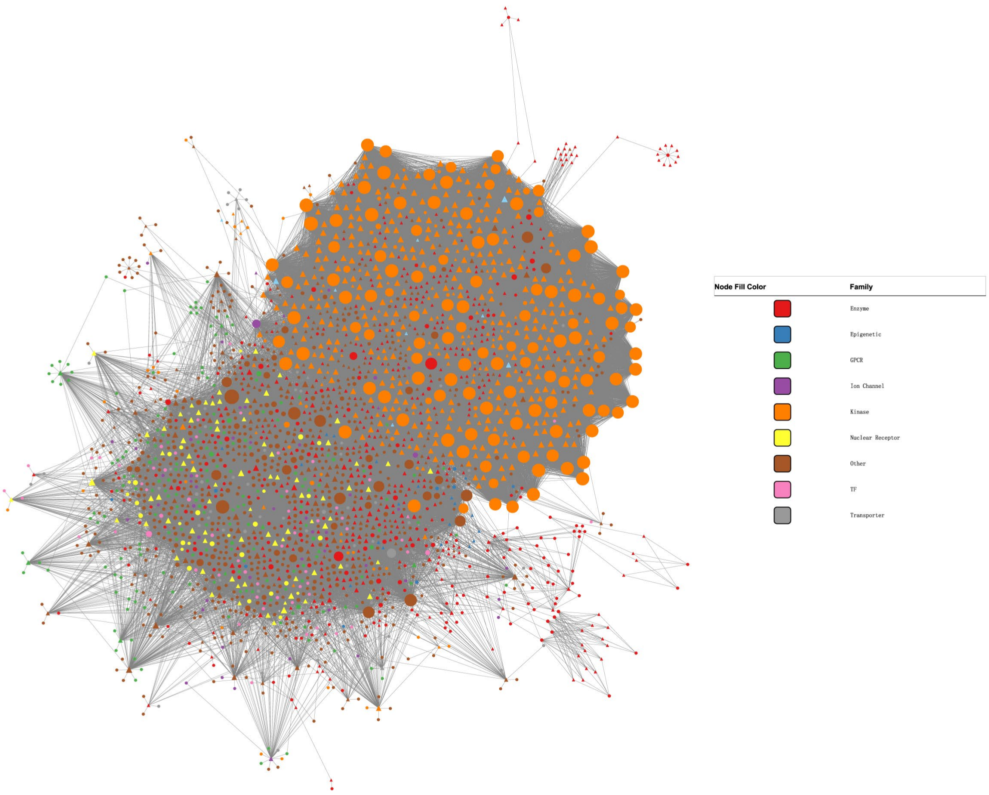

Figure S4. Network of similar pocket pairs between druggable pockets in the human proteome and drug molecule pockets. Triangles represent drug molecule pockets, and circles represent druggable pockets in the human proteome .
